# Mcleod syndrome: Report of an Indian family with phenotypic heterogeneity

**DOI:** 10.4103/0972-2327.78053

**Published:** 2011

**Authors:** Ambar Chakravarty, P. Bhattacharya, D. Banerjee, S. Mukherjee

**Affiliations:** Department of Neurology and Hematology, Vivekananda Institute of Medical Sciences, Kolkatta, India; 1 Department of Transfusion Medicine and Hematology, AMRI Hospitals, Kolkatta, India

**Keywords:** Anterior horn cell, McLeod syndrome, XK gene

## Abstract

The present report deals with the clinical phenomenology of three members (brothers) of one family with McLeod syndrome (MLS). In two, the clinical pictures were of choreiform disorders with amyotrophy, which were found to be neurogenic in origin by detailed electrophysiological study. The index case had peripheral acanthocytosis; immunohematological and molecular genetic studies confirmed diagnosis of MLS. However, one brother only had a slowly progressive motor neuron disease like picture but no abnormal movement disorder. He had peripheral acanthocytes as well. The inheritance seems to be X-linked recessive in nature. The affected family members exhibited much phenotypic heterogeneity. This appears to be the first report of MLS from India.

## Introduction

McLeod syndrome (MLS) is an X-linked recessive multisystem disorder that is generally classified along with neuroacanthocytosis syndromes.[1] The characteristic feature of MLS is the absence of Kx RBC antigen, weak expression of Kell RBC antigens, acanthocytosis, and compensated hemolysis. Asymptomatic carriers may be detected during blood bank testing.[2,3] Carriers have elevated CK levels. Neurological signs and symptoms develop with a mean age of 30–40 years (range 25–60 years).[3–5] Neuromuscular manifestations include myopathy, sensory-motor axonal neuropathy, and cardiomyopathy. Central nervous system (CNS) manifestations include choreic movement disorders, subcortical cognitive impairment, psychiatric abnormalities, and generalized seizures. MLS is caused by mutations of the XK gene encoding the XK protein, which carries the Kx RBC antigen;[6] the exact function of the gene being unknown. Judging by clinical features of MLS patients, it is possible that the gene may not only be involved in RBC physiology but may also be involved in apoptosis regulation, disturbance of which causes neurodegeneration. MLS has been recently reviewed by Jung *et al*[7] and Jung.[8] We report here the clinical features of three members of a family where the index case had been fully investigated using immunohematological and molecular genetic aspects confirming the diagnosis of MLS. This is to our knowledge the first report of a MLS family from India.

## Case Reports

### Case 1 (Index case)

This 53-year-old-man first presented in October 2008. He was born of non-consanguineous parentage and is the youngest of three brothers whose neurological features are also detailed in this article. His parents died of cerebrovascular disease and had no prior history of any neurological disease. Also, as far as could be elicited, there was no history of any inherited neurologic disease in the family except in the present generation. He was well until the age 50 years when he first noticed frequent muscle cramps even at rest. This was soon followed by development of abnormal involuntary movements involving all four limbs and orolingual muscles. These symptoms were progressive and he developed slurring of speech and marked abnormal movements of the tongue and perioral muscles. He started biting his tongue and lower lip. Along with these abnormal movements, he developed weakness of the upper and lower limb muscles with difficulty in walking. On examination, his cognitive function appeared normal (MMSE 30/30), he had a slurred speech, and frequent tongue protrusions were noted. The tongue and lower lip bore injury marks. There were choreiform movements of all four limbs but no rigidity. His gait was unsteady as a combined effect of weakness and abnormal movements. There was significant asymmetrical (L> R affected) weakness of all four limbs. Hand grips were weak but there was no small muscle wasting. The proximal limb muscles were however wasted and occasional fasciculations detected in the deltoid and triceps. Tendon reflexes were all absent and planters were flexor. There was no sensory change including vibration and proprioception. He denied video photography.

His MR brain scan revealed mild atrophy of the caudate nucleus head. EMG showed spontaneous activity at rest (fibrillations, fasciculations, and positive sharp waves), and poor recruitment of high amplitude long duration potentials in affected muscles and also in hand muscles. The findings suggested chronic partial denervation with re-innervation as seen in diffuse anterior horn cell disease. Nerve conduction studies showed only mild slowing in upper and lower limb nerves with reduced amplitude of CMAP. Sensory conductions were normal. His serum CK was mildly raised (400 units; normal up to 180 units; MB/MM fractions not done) and serum lipid profile and lipoprotein electrophoresis revealed normal values. Serum ceruloplasmin was normal. His ECG, Holter monitoring, and echocardiography were normal. Consent for muscle biopsy was denied.

**Figure 1:**
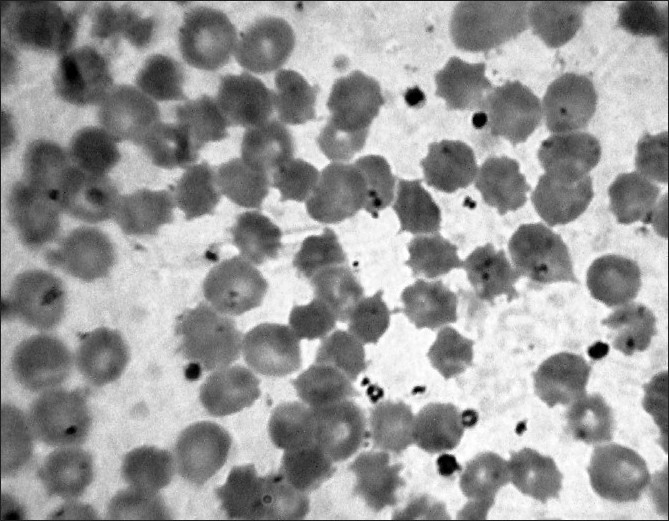
Peripheral blood film of Case 1 showing acanthocytes (×1000)

Hematological and molecular investigations: His peripheral smear examination with Leishman stain showed an abnormally high percentage of acanthocytes red cells with spiny projections from the cell membrane (75%)(done by PB and DB). No special procedures for demonstration were needed [Figure 1].

Red cell serological analysis of his blood group showed O Rh (D) Positive. Extended Rh and Kell phenotype showed C+,c+,E-,e+,K-(Dia Clon, Dia Med, lot number, 50110.73.02). His direct antiglobulin test (DAT) was negative and extended blood group antigen profile typing showed k-, Kpa-, Kpb-, Jka+, Jkb- (DiaMed lot number, 5038.43.01) (done by PB and SM).

Results of red cell adsorption and elution studies indicated weaker expression of the k (Cellano) antigen.

The clinical features and investigation results in this male patient, with progressive neurodegeneration, acanthocytes, and weak expression of Kell antigen, highly indicated Mcleod phenotype.[2]

Molecular study of XK gene (with informed written consent) was detailed at International Blood Group Reference Laboratory (NHS Blood and Transplant) Bristol, UK, which revealed deletion of exon 2; however, exon 1 and exon 3 on PCR amplification confirmed no mutations. This had been reported earlier by the same group.[9]

Course: His choreiform movements and tongue dyskinesia significantly improved following treatment with Tetrabenazine 25 mg three times daily (TID) and he is still working under this regimen.

### Case 2

This 56-year-old-man, elder brother of the index case, was bed ridden when first presented to the author. He had a history of having had poliomyelitis at the age of 4 years and was left with a slightly wasted left leg but was in no way disabled. At the age of 35 years, he first noticed weakness in both legs with progressive difficulty in walking. Some years later he also noticed weakness in both upper limbs and had difficulty in raising his arms above shoulder. Over the years his disability increased but he never developed any form of abnormal movements. A CT scan of his brain was normal. His EMG (done on 3 occasions) was suggestive of a diffuse anterior horn cell disease with spontaneous activity at rest and poor recruitment with neuropathic potentials on volition. Nerve conduction study showed reduced CMAP amplitude, prolonged distal latency and slow conduction velocity in lower limbs and upper limbs suggestive of axonal degeneration. Sensory conductions were absent in upper limbs. Over the years, his clinical diagnosis varied from post-polio syndrome to Motor neuron disease and he was treated with Riluzole for a while.

When examined in February 2009, he was bed ridden with slurred speech and dysphagia. His MMSE score was 28/30. His external ocular and sphincteric functions were intact. There were no abnormal involuntary movements anywhere. The tongue was wasted and showed fibrillations. There was gross weakness and wasting of all four limbs with occasional fasciculations in the upper limbs. He had total tendon areflexia.

His serum CK was normal (166 units) and a freshly drawn blood smear showed plenty (>50%) acanthocytes (Leishman stain) (DB). Electrophysiology was repeated. EMG features suggested evidence of chronic partial denervation with re-innervation and nerve conduction studies were suggestive of axonal neuropathy. Consent for immunohematology, molecular genetic, and muscle histology studies was not obtained. ECG, Holter monitoring, and echocardiography were normal.

### Case 3

The eldest of the three brothers died several years back when he was aged 45 years. His medical records were not available. According to close relatives, he developed abnormal limb movements and orolingual dyskinesia with tongue and lip injury like the index case (Case 1) in his mid thirties. These progressed and later he developed weakness of all four limbs. He also had generalized seizures. His walking deteriorated over the years and he was bed ridden for the last 2 years of his life. He probably died of sepsis. It is not clear whether he had any cognitive deficiency.

## Discussion

The diagnosis of McLeod syndrome (MLS) in the index case was confirmed by immunohematological and molecular genetic studies. Indeed, these could not be performed in Case 2. However in view of the family history, MLS appeared to be the most likely diagnosis in Case 2 who also showed peripheral acanthocytosis, and also in Case 3 in view of the closeness of clinical features to those of the index case. The ages of onset in the elder two siblings were around 35 years whereas in the younger (index case) it had been later (50 years) There are three interesting features in relation to neurological features in the family. The family showed much phenotypic heterogeneity in the sense that the index case and Case 3 showed combination of choreiform movement disorder with a neuromuscular syndrome, whereas Case 2 only had a neuromuscular syndrome and no movement disorder. Phenotypic heterogeneity had been recorded in MLS earlier.[10] Second, tongue dyskinesia is uncommon in MLS and more common in chorea-acanthocytosis. In Case 1, the diagnosis of MLS is confirmed by genetic analysis. Last, the neuromuscular syndromes in both the index case and Case 2 were neurogenic in origin as suggested by electrophysiology and seemed suggestive of anterior horn cell involvement (as in motor neuron disease), most likely. This notion was also supported by clinical observation of limb and tongue fasciculations. The possibility of an associated motor axonopathy could not be excluded altogether. This is somewhat different from the classical neuromuscular manifestation in MLS, which is usually a myopathy.[7] However, both myopathic and neuropathic features in muscle histology have been reported in MLS.[3] In a recent study, Hewer *et al*[11] demonstrated neuropathic changes in muscle histology in all ten patients of their series and ascribed them to motor axonopathy. In the index case of the present study, clear neuropathic changes were seen in EMG but no electrophysiological evidence of axonal neuropathy was noted. In Case 2, however, evidence of axonal neuropathy was evident. We therefore conjectured on involvement of spinal AHC at least in the index case. The raised CK in this case may however suggest an associated myopathy as well. Although brain pathology had been reported in MLS,[7] we are not sure whether the spinal cords had been examined. Basal ganglionic atrophy had been noted. Rinne *et al*[15] in a neuropathological study on autopsied brains and spinal cords of three patients with neuroacanthocytosis (not MLS), noted involvement of anterior horns with neuronal loss and gliosis. Case 3 probably had seizures but neither of the two cases examined showed any evidence of cognitive deficit. None of the patients examined had cardiomyopathy clinically or by ECG and echocardiography. MLS is a rare syndrome. Although the population prevalence had been estimated to vary from 0.5–1 per 100,000 subjects,[12] till 2001, only 60 proven cases of MLS are on record.[3] This suggests that MLS is grossly under diagnosed. We would suggest routine screening for Kx RBC antigen during blood grouping as also testing for Kell antigen in all neurological patients with peripheral smear acanthocytes. Patients presenting with a cardiomyopathy need to be screened as well.[13,14]
